# Underreported and unknown student harassment at the Faculty of Science

**DOI:** 10.1371/journal.pone.0215067

**Published:** 2019-04-25

**Authors:** Laura Jussen, Toine Lagro-Janssen, Joke Leenders, Colin Logie, Rachel Mijdam

**Affiliations:** 1 Gender and Diversity Committee of the Faculty of Science of the Radboud University, Nijmegen, The Netherlands; 2 Dept. of Primary and Community Care, Radboud University Medical Centre, Nijmegen, The Netherlands; 3 Nijmegen School of Management, Nijmegen, The Netherlands; Northwestern University, UNITED STATES

## Abstract

Reports of sexual harassment at medical faculties throughout the world, including the Radboud University, raised the question how prevalent this is at the Faculty of Science. We performed a survey among students to assess their experiences with harassment. This questionnaire consisted of questions from the EGERA survey, a questionnaire held among staff of multiple European Universities. We found that 9% of the respondents had observed or experienced harassment at the Faculty. Hardly any of these cases were reported to one of the institutional services. Moreover, most students did not now any of the provided services. We therefore suggest raising awareness on harassment and to make students more familiar with the trust person.

## 1. Introduction

In 2005, the Dutch Journal for Medical Sciences (‘Nederlands Tijdschrijft voor Geneeskunde’) reported on sexual harassment as a large problem among medical interns in the Netherlands [1]. Since then, multiple studies have researched the extent and degree of sexual harassment at medical faculties. Over the years nothing changed, despite the efforts to make sexual harassment discussable and easier to report. Still almost 20% of medicine students and medical interns felt sexually intimidated [2]. At other universities throughout the USA and Europe this rate was even higher at 30–50% [3]. Among offenders of medical students and medical interns were staff members, teachers, nurses and patients [1, 2]. According to a large investigation, sexual harassment is a widespread problem among universities, not only at Faculties of Medicine, but at Faculties of Engineering and Science as well [4].

Harassment can affect the mental and physical health of students and their academic engagement, even observers who are not part of the targeted group. Not only immediately after the incident, but also in the long term [5]. One of the most important mental consequences of sexual harassment, underlying depression and anxiety disorders, is a post-traumatic stress disorder (PTSD). A PTSD can, if not treated well, last for years with an impact on the quality of life and on graduates’ values and behaviour in their future working life [6]. Academic institutions should therefore prevent harassment where possible.

The frequency of sexual intimidation at medical faculties raised the question: How prevalent is this at our Faculty? We hypothesized that, based on literature, students in the Faculty of Medicine will report the greatest amount of harassment, more commonly also than those in the Faculties of Science and Technology [7]. Nevertheless, students of all faculties reported harassment.

The Faculty of Science of the Radboud University is a student-oriented Faculty where research and education are closely connected [8]. Students and employees from different backgrounds work closely together. In the academic year 2016/2017 the total number of students at was 2271 and there were 573 Full Time Equivalent (FTE) of academic staff and 285 FTE of supporting staff [9]. In 2015 and 2017, a survey on organization culture was distributed among academic staff at the Radboud University in the context of EGERA (Effective Gender Equality in Research and the Academia), a study funded by the European Union. This survey involved questions about harassment among staff members [10]. Harassment was defined as "unwanted conduct occurring with the purpose or effect of violating the dignity of a person and of creating an intimidating, hostile, degrading, humiliating or offensive environment" [11]. However, no research on sexual harassment or harassment in general had been performed before at this faculty among students. Therefore, we complemented the EGERA research by performing a survey among students to assess their experiences with harassment.

## Materials and methods

To obtain more insight into the prevalence of harassment at the Faculty of Science at Radboud University, a questionnaire was sent to all of its 2271 Bachelor’s and Master’s students. The working group ‘Intimidation’ of the Faculty’s Gender and Diversity committee (https://www.ru.nl/science/about_the_faculty/our-profile/gender-diversity-policy/) prepared the questionnaire (S1 File) and on-line survey. Students were invited to participate by the Faculty Board in an e-mail in June 2017 (S2 File). The invitation was both in Dutch and English, although the questionnaire itself was only in English (S3 File).

Participants knew the questionnaire was anonymous, as it was said in the invitation. We sent the questionnaire to all students of the Faculty via the student alias, so no names were visible. The results were shown to us in the program Lime Survey, which only shows the answers of the participant but not their names.

The questions were selected from the EGERA project questionnaire. These questions have been used before in European connection with a good feasibility [10]. The questions comprised background information on the respondent, whether they had experienced or observed any harassment, on basis of what characteristics it occurred and by whom, and whether they had reported it. Further, to get a better view on the range of harassments we asked for an example of the case. Participants could only choose from predefined answers, except for the question where they were asked to give examples. Respondents could choose characteristics for which they had experienced or observed different kinds of harassment. The harassment categories used in the EGERA-survey are: physical, psychological, verbal and sexual. These forms of harassment could be targeted on different predefined characteristics. The institutional services where students could report their experiences and observations or find more information were selected from the EGERA project questionnaire. These services included the trust person, prevention advisor, internal claims procedure against violence, bullying and harassment, regulations on undesirable behaviour, complaints committee for undesirable behaviour and the confidential advisor.

Only descriptive statistics were performed since the numbers were small and it was the first time this questionnaire was held. Statistical analysis was performed using SPSS and Excel.

The need for ethical approval of this study was waved by the Ethics Assessment Committee of the Faculty of Law and Nijmegen School of Management.

## Results

### Survey participants

**Fig 1:**
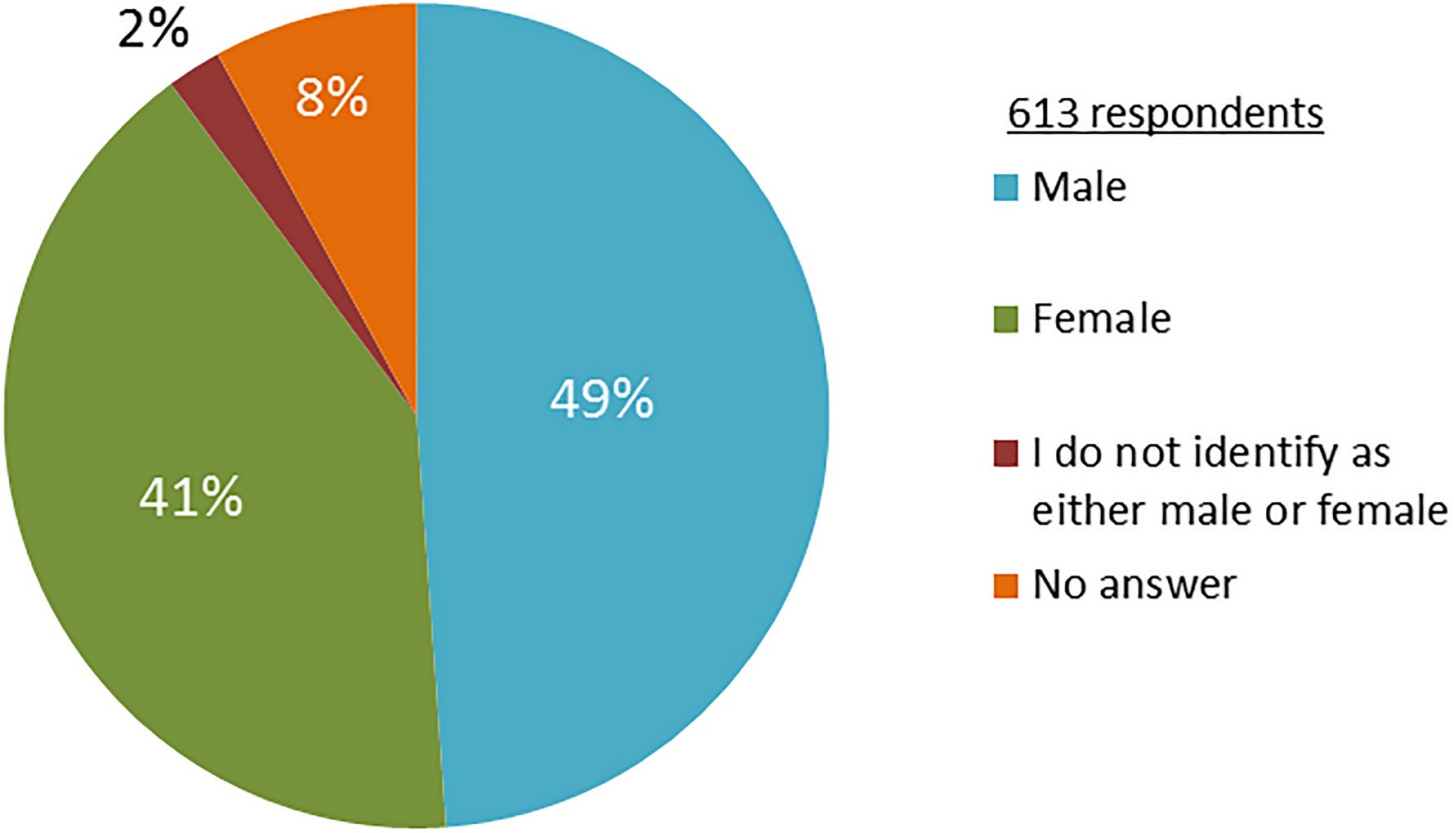
Gender of respondents. Pie chart of the declared gender of the responding students.

The Faculty of Science counted 37% female and 63% male students in June 2017. Of these 2771 students, 613 started the questionnaire (22%) and 518 completely filled in the complete questionnaire. The answers of three respondents were removed because they did not fill in the questionnaire properly. The remaining 610 respondents were from all years (S1 Table) and all Science Bachelor and Master studies were represented (S2 Table). Students can follow two Bachelors. Of the respondents, 49% declared male and 41% female, 2% did not identify as either male or female and 8% did not answer the question regarding their gender identity (Fig 1). In June 2017 there were 10% foreign students. The survey was mostly answered by Dutch students (481, 79%), followed by German students (23, 4%) and Spanish students (5, 1%); 70 students did not mention their nationality (11%) (S3 Table).

### Experienced and observed harassment

**Fig 2:**
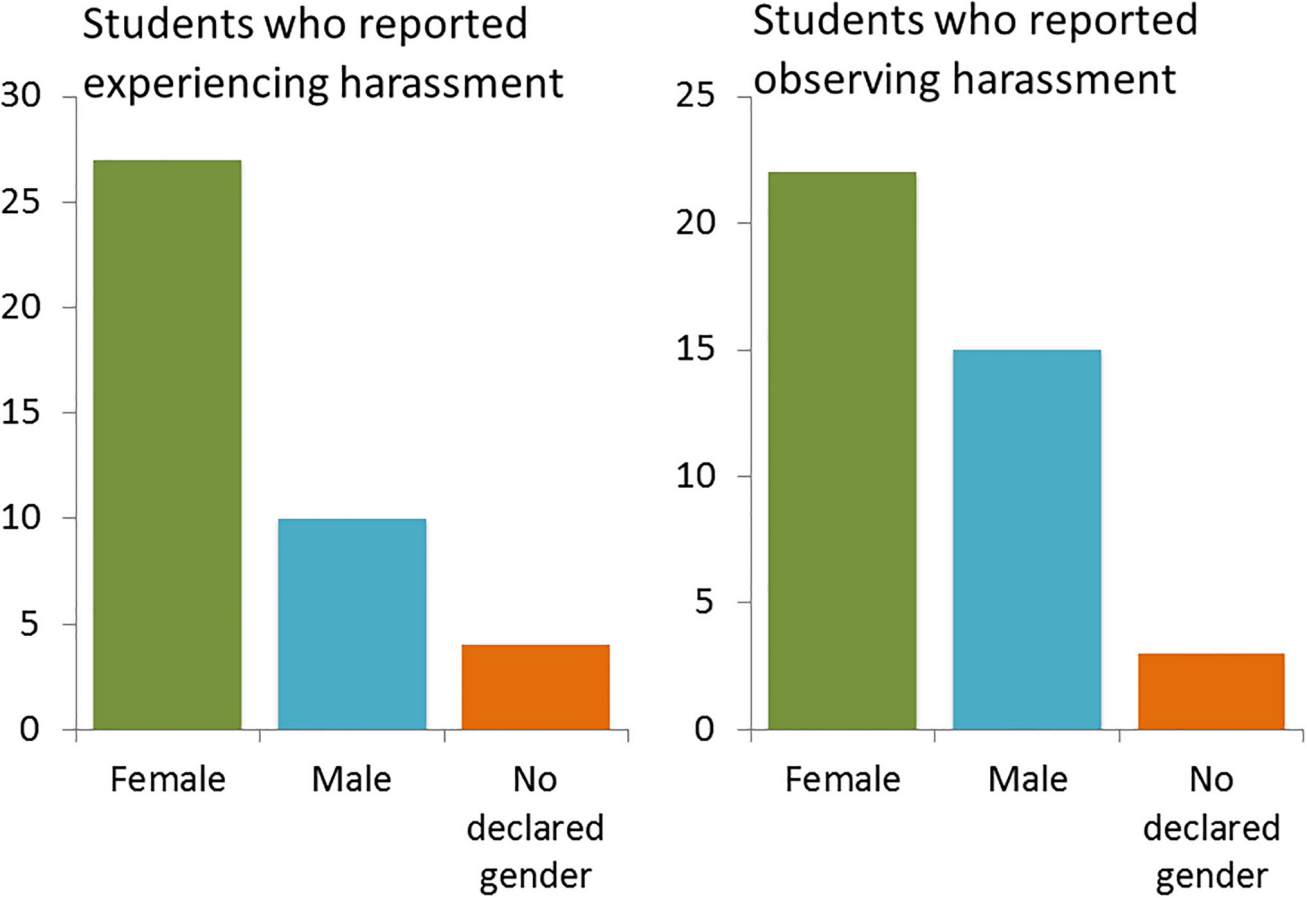
Experienced and observed student harassment. Number of students who have experienced (left panel) or observed (right panel) harassment at the Faculty of Science, stratified according to their declared gender.

**Fig 3:**
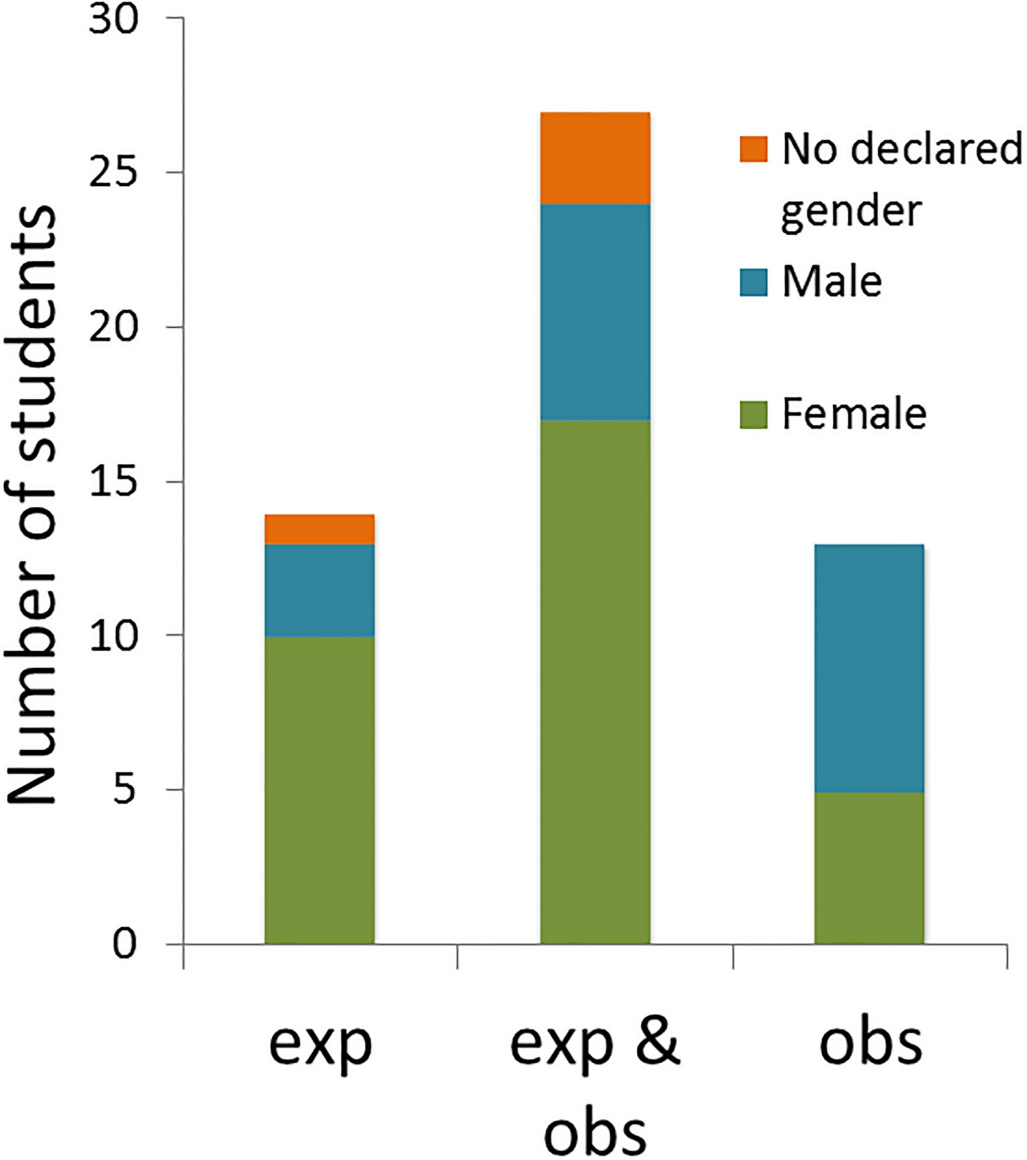
Overlap between experienced and observed harassment. Number of students that reported only observing, both observing and experiencing or only experiencing harassment. Data are stratified according to declared gender.

**Fig 4:**
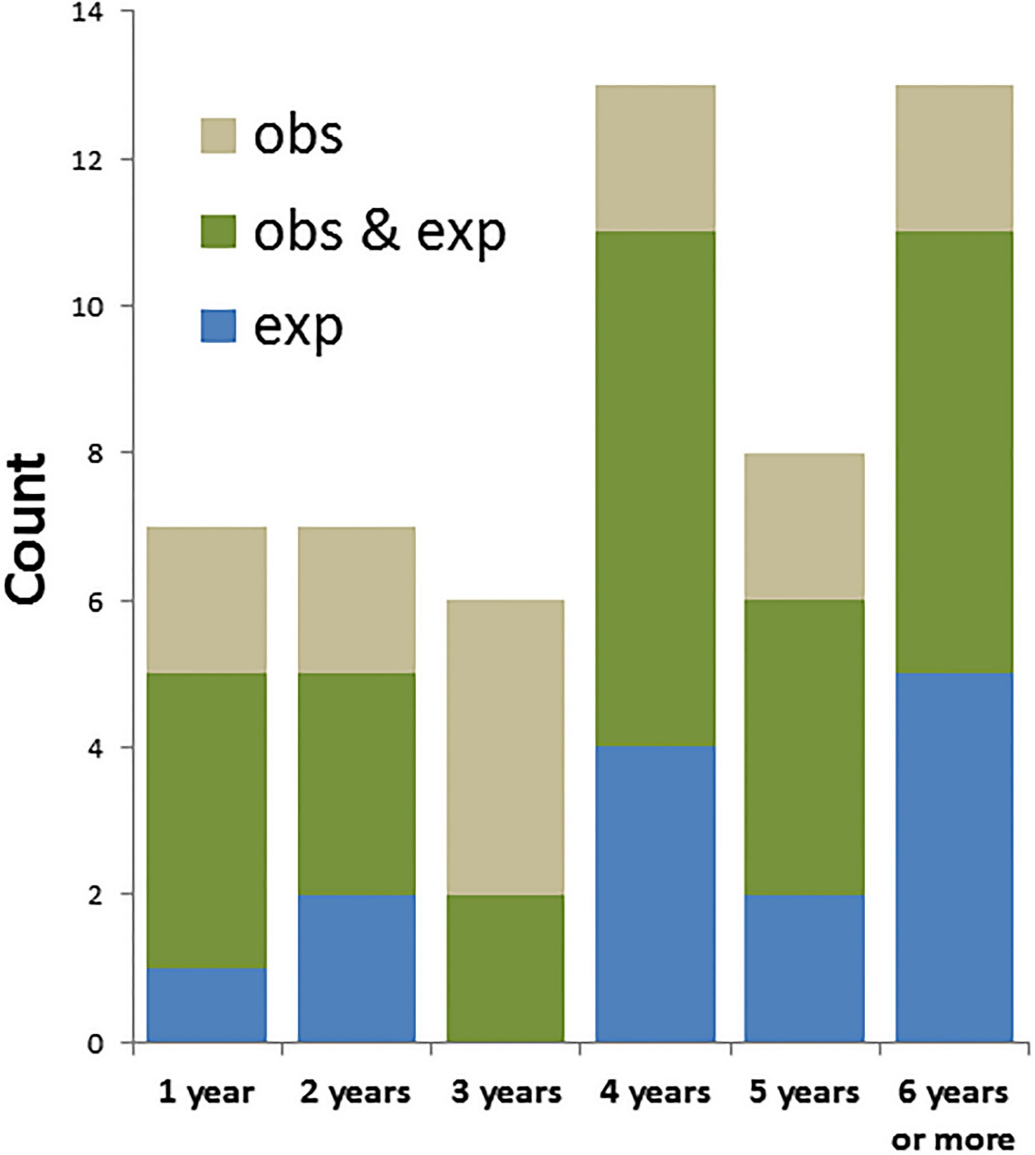
Study time versus harassment observation and experience. Number of observations and/or experiences are plotted as a function of the number of years respondents had been studying at the Faculty of Science.

Students were asked whether they have experienced or observed harassment. Overall, 54 respondents (9% of all respondents) reported observing or experiencing harassment at the Faculty. Of all respondents, 41 students (6.7%) had experienced harassment, of these students 24% was male, 67% female and 9% identified other (Fig 2). In total, 40 students (6.5%), of which 38% was male, 55% female and 8% identified other, had observed harassment at the Faculty of Science (Fig 2). These experiences and observations have been throughout the entire time respondents had been studying at the Faculty of Science. Notably, 27 students (50%) experienced *and* observed harassment (Fig 3). Respondents who had studied four or more years reported more harassment (Fig 4), as would be expected since they spent more time at the Faculty.

Psychological and verbal harassment were the most experienced forms of harassment (both 35%), followed by physical harassment (17%) and sexual harassment (12%). Observers saw mostly psychological harassment (36%) and verbal harassment (35%), followed by physical (17%) and sexual harassment (13%). The characteristics ‘sex’ and ‘physical appearance’ led to the most harassment (S6 and S7 Tables), particularly for female students. Of harassment on ‘my sex’ 86% was experienced by females and on ‘my physical appearance’ was experienced by 79% females. Students experienced and observed multiple forms of harassment, since the total number of different kinds of harassment named by respondents (158 for experienced and 132 for observed harassment) is much higher than the number of students who had experienced or observed harassment. Examples of other reasons for harassment can be found in S8 Table.

We asked students to indicate how often they have experienced or observed harassment and by whom. The perpetrators of harassment that were identified were supervisors, students and other employees of the Faculty (S4 and S5 Tables).

“*For her*, *these people were a reason to leave the university*. *It was one student and one supervisor in our first weeks at university*, *and they were saying that if she isn’t able to speak properly*, *she maybe isn’t able to do anything in the lab or is too stupid*. *That she should go back*. *For me this was the worst I saw in the Netherlands in all these years till now*. *Otherwise everyone is really really kind and helpful*.”

### Reporting harassment

Of the students who had experienced harassment 74% answered the question on reporting harassment, only 4 of these students (13%) reported their experience to the provided institutional services, while 27 students (87%) did not. Of the students who had observed harassment, 34 (85%) answered the question on reporting harassment, 5 (15%) reported it to the provided institutional services, 29 (85%) did not. Reasons to not report the harassment are e.g. not knowing where to report it, fighting the problem themselves, the wish of the harassed to not report it, or thinking reporting it will not make a difference (S8 Table).

We asked students to indicate which services at the Faculty are known to them. The trust person was known by 212 students (35%), the prevention advisor by 25 students (4%), the internal claims procedure against violence, bullying and harassment by 32 students (5%). Conversely, 304 students (50%) indicated that they did not know any of these services. The regulations on undesirable behaviour were known by 65 students (11%), the complaints committee for undesirable behaviour by 39 students (6%), the confidential adviser by 133 students (22%). Conversely, 353 students (58%) did not know any of these services.

“*They said I would get into trouble if I told anyone*.”

## Discussion

Our study shows that 7% of the respondents (n = 41) reported experiencing harassment, representing 1.8% of all potentially surveyed students. In absolute numbers, 20 of these (± 2% of all female students) have reported sexual harassment events, ranging from once (2 occurrences) to often (6 occurrences). The actual number of students who have experienced harassment might be higher, since non-responders could also have experienced harassment but were not registered through this survey, which may lead to underreporting.

As inherent for a harassment questionnaire, and despite using a definition of harassment that encompasses the notion of ‘unwanted conduct’ [11], inherent subjectivity as to the boundaries for harassment and the assessment of its degree are dictated by each respondent. This limitation makes the results difficult to quantify, and strict guidelines difficult to design. However, these limitations are not limited to this report or this Faculty but part of the field.

Harassment is experienced and observed by both male and female students. In accordance to literature female students more commonly report harassment of all categories than males [4, 7, 11]. The male response to the questionnaire of 48%, allows us to state that harassment is experienced and observed by both male and female students. However, for declared males, all the experienced harassment was of a non-sexual nature. The finding that in our survey none of the male students mentioned sexual harassment is noteworthy. This profound dearth of male students reporting sexual harassment therefore appears to be a gender dimorphism. It is an open question as to whether this is at the level of true experience, insensitivity to the situation, or under-reporting. In other investigations it has been found as well that women are sexually harassed more often than men [4]. This does raise the question whether this might suggest that measures should not only be unisex, but could also be specified for women and men since the problem is gendered.

Although the data of the respondents gives us an insight in the prevalence of harassment at the Faculty of Science, we must note that only 613 (22%) of the total 2771 students started the questionnaire and 518 (19%) filled in the questionnaire completely. We cannot assess the prevalence of victims and witnesses amongst the 78% of students who did not respond to our survey. In 610 acceptable questionnaires, there are 41 reports of harassment experience and 40 of harassment observation. The two types are very congruent when stratified in categories. About 20% of occurrences are categorized as sexual, and 34 to 37% verbal or psychological. Physical harassment was more experienced (29%) than observed (20%).

According to our results, it seems that students who have experienced harassment are also more likely to observe it. This might suggest that own experience makes them more aware of harassment in their surrounding or vice versa: when students have observed harassment, they might recognize it more easily when it happens to them.

Our findings are much lower than the percentage of harassment seen at the Medical Faculty. This gives the impression that it is going well. However, arguably every single case of harassment is worrisome. We should keep reducing harassment as much as we can, even though very few cases of harassment have been reported to the provided institutional services. Even when people experience or observe harassment they are reluctant to confront perpetrators [12]. A reason for this might be that harassment is a difficult topic to speak about. People tend to keep their story to themselves and think that speaking up will only make the problem bigger. However, in literature it is found that it is better to share your experience [13]. On the other hand, very few students know about the trust advisor and other services. Therefore, they may simply not know where to report the experience or observation.

“*The trust person took me seriously*, *that felt good*”

### General recommendations made to the Science Faculty on the basis of the present survey

At the human level, the availability of a diverse set of visible interlocutors (men, women and identified minorities) is likely to be a plus to lower the threshold for reporting. Furthermore, to accomplish higher levels of harassment reporting, awareness of harassment reporting modalities needs to be raised in the students, lecturers and supporting personnel of the Faculty.

The EGERA questionnaire, and its present adaptation for students can mechanically improve reporting rates because it represents a partial answer to the needed for simplification of reporting through virtual reporting points that are centralised in an efficient though ethically acceptable fashion. We therefore suggest to repeat the questionnaire on a bi-yearly basis to monitor future developments and observe student cohorts longitudinally.

## Supporting information

S1 File On-line survey questionnaire. (PDF)

S2 File Faculty Board invitation to participate in the survey emailed in June 2017. (PDF)

S3 File Lime survey provided as a .lss file. (LSS)

S1 Table Number of years respondents have been studying. (DOCX)

S2 Table Programs followed by respondents. (DOCX)

S3 Table Nationality of respondents. (DOCX)

S4 Table Experienced harassment of students by supervisors, students and other employees of the Faculty. (DOCX)

S5 Table Observed harassment of students by supervisors, students and other employees of the faculty. (DOCX)

S6 Table Experienced physical, psychological, verbal and sexual harassment according to the characteristics. (DOCX)

S7 Table Observed physical, psychological, verbal and sexual harassment according to the characteristics. (DOCX)

S8 Table Examples of free text comments. (DOCX)
