# Supplementary material for: Underreported and unknown student harassment at the Faculty of Science
Source: PLoS One. 2019 Apr 25;14(4):e0215067. doi: 10.1371/journal.pone.0215067 (PMC6483172; doi:10.1371/journal.pone.0215067)
Supplement: S1 File — (PDF) [file pone.0215067.s001.pdf]

Supplementary File Questionnaire

General questions

1. How long have you been studying at the Faculty of Science?
    a. 1 year
    b. 2 years
    c. 3 years
    d. 4 years
    e. 5 years
    f. 6 years or more

2. Which Bachelor's or Master's programme do you follow?
    a. Bachelor Biologie
    b. Bachelor Informatica
    c. Bachelor Molecular Life Sciences
    d. Bachelor Natuur- en Sterrenkunde
    e. Bachelor Science
    f. Bachelor Chemistry
    g. Bachelor Wiskunde
    h. Master Biology
    i. Master Medical Biology
    j. Master Molecular Life Sciences
    k. Master Chemistry
    l. Master Science
    m. Master Physics and Astronomy
    n. Master Mathematics
    o. Master Computing Science
    p. Master Information Sciences

3. What is your gender?
    a. Male
    b. Female
    c. I do not identify as either male or female

4. What is your nationality?
    a. Open answer

Harassment

5. Harassment is defined as "unwanted conduct occurring with the purpose or effect of violating the dignity of a person and of creating an intimidating, hostile, degrading, humiliating or offensive environment." Have you experienced harassment at the faculty?
    a. Yes
    b. No

6. Have you observed any harassment at the faculty?
    a. Yes
    b. No

7. According to your answers, you have experienced harassment at the faculty. Could you please indicate for which characteristic(s) you have been the target of <u>physical</u> harassment?

a. My sex
b. My age
c. My skin colour
d. My ethnic cultural original
e. My physical appearance
f. My sexual orientation
g. My disability
h. The way I speak
i. My political ideas
j. My religion or philosophy
k. My social class
l. My native language
m. Unknown reasons
n. Other: Open answer

8. According to your answers, you have experienced harassment at the faculty. Could you please indicate for which characteristic(s) you have been the target of <u>psychological</u> harassment?
   a. My sex
   b. My age
   c. My skin colour
   d. My ethnic cultural original
   e. My physical appearance
   f. My sexual orientation
   g. My disability
   h. The way I speak
   i. My political ideas
   j. My religion or philosophy
   k. My social class
   l. My native language
   m. Unknown reasons
   n. Other: Open answer

9. According to your answers, you have experienced harassment at the faculty. Could you please indicate for which characteristic(s) you have been the target of <u>verbal</u> harassment?
   a. My sex
   b. My age
   c. My skin colour
   d. My ethnic cultural original
   e. My physical appearance
   f. My sexual orientation
   g. My disability
   h. The way I speak
   i. My political ideas
   j. My religion or philosophy
   k. My social class
   l. My native language
   m. Unknown reasons
   n. Other: Open answer

10. According to your answers, you have experienced harassment at the faculty. Could you please indicate for which characteristic(s) you have been the target of <u>sexual</u> harassment?
    a. My sex
    b. My age
    c. My skin colour
    d. My ethnic cultural original
    e. My physical appearance
    f. My sexual orientation

g. My disability
h. The way I speak
i. My political ideas
j. My religion or philosophy
k. My social class
l. My native language
m. Unknown reasons
n. Other: Open answer

11. Could you please indicate how often you have experienced harassment at your institution and by whom?
    a. Supervisor (Lecturer, thesis supervisor, mentor etc.): Once/Seldom/Regularly/Often/Not applicable/No answer
    b. Students: Once/Seldom/Regularly/Often/Not applicable/No answer
    c. Any other employee of the faculty: Once/Seldom/Regularly/Often/Not applicable/No answer

12. Did you report one or more of your experiences with harassment to (one of the) provided institutional services?
    a. Yes
    b. No. Can you give the reason(s) why you did not report your experience(s)? Open answer

13. If you would like to add further information on your experiences with harassment, you can do so below:
    a. Open answer

14. According to your answers, you have observed harassment at the faculty. Could you please indicate for which characteristic(s) you have been the target of <u>physical</u> harassment?
    a. Their sex
    b. Their age
    c. Their skin colour
    d. Their ethnic cultural original
    e. Their physical appearance
    f. Their sexual orientation
    g. Their disability
    h. The way they speak
    i. Their political ideas
    j. Their religion or philosophy
    k. Their social class
    l. Their native language
    m. Unknown reasons
    n. Other: Open answer

15. According to your answers, you have observed harassment at the faculty. Could you please indicate for which characteristic(s) you have been the target of <u>psychological</u> harassment?
    a. Their sex
    b. Their age
    c. Their skin colour
    d. Their ethnic cultural original
    e. Their physical appearance
    f. Their sexual orientation
    g. Their disability
    h. The way they speak
    i. Their political ideas
    j. Their religion or philosophy
    k. Their social class
    l. Their native language
    m. Unknown reasons

n. Other: Open answer

16. According to your answers, you have observed harassment at the faculty. Could you please indicate for which characteristic(s) you have been the target of verbal harassment?
    a. Their sex
    b. Their age
    c. Their skin colour
    d. Their ethnic cultural original
    e. Their physical appearance
    f. Their sexual orientation
    g. Their disability
    h. The way they speak
    i. Their political ideas
    j. Their religion or philosophy
    k. Their social class
    l. Their native language
    m. Unknown reasons
    n. Other: Open answer

17. According to your answers, you have observed harassment at the faculty. Could you please indicate for which characteristic(s) you have been the target of sexual harassment?
    a. Their sex
    b. Their age
    c. Their skin colour
    d. Their ethnic cultural original
    e. Their physical appearance
    f. Their sexual orientation
    g. Their disability
    h. The way they speak
    i. Their political ideas
    j. Their religion or philosophy
    k. Their social class
    l. Their native language
    m. Unknown reasons
    n. Other: Open answer

18. Could you please indicate how often you have observed harassment at your institution and by whom?
    a. Supervisor (Lecturer, thesis supervisor, mentor etc.): Once/Seldom/Regularly/Often/Not applicable/No answer
    b. Students: Once/Seldom/Regularly/Often/Not applicable/No answer
    c. Any other employee of the faculty: Once/Seldom/Regularly/Often/Not applicable/No answer

19. Did you report one or more of your observations of harassment to (one of the) provided institutional services?
    a. Yes
    b. No. Can you give the reason(s) why you did not report your experience(s)? Open answer

20. If you would like to add further information on your observations of harassment, you can do so below:
    a. Open answer

21. Could you please indicate which of the following services at the faculty are known to you?
    a. Trust person
    b. Prevention advisor
    c. Internal claims procedure against violence, bullying and harassment
    d. None of the above

22. Could you please indicate which of the following services at the faculty are known to you?

a. Regulations on undesirable behaviour
b. Complaints committee for undesirable behaviour
c. Confidential adviser
d. None of the above
