# Supplementary material for: Underreported and unknown student harassment at the Faculty of Science
Source: PLoS One. 2019 Apr 25;14(4):e0215067. doi: 10.1371/journal.pone.0215067 (PMC6483172; doi:10.1371/journal.pone.0215067)
Supplement: S2 File — (PDF) [file pone.0215067.s002.pdf]

Supplemental file 2; Invitation email sent on June 12th, 2017

[Dutch follows English]

Dear student,

The Faculty of Science wants everyone to feel welcome. Recently, there has been a survey among employees to investigate whether they experience harassment here.

So far, there is no information about students on this issue. Therefore, the Gender And Diversity Committee invites you to fill in this questionnaire.

It takes max. 5 minutes. You can also write your answers in Dutch.

This questionnaire is anonymous!

If you would like to talk to somebody about the issues raised in this questionnaire, please contact the confidential advisors at the Faculty of Science (Name, telephone number, email@address; Name 2, telephone number, email@address).

Thank you for your help!

On behalf of the workgroup harassment, Gender and Diversity Committee

---

Beste student,

De faculteit NWI wil graag dat zich iedereen welkom voelt. Onlangs is er een enquête geweest onder medewerkers om te inventariseren of ze hier intimidatie ervaren.

Tot nu toe is er geen informatie beschikbaar over de ervaringen van studenten op dit gebied. Daarom nodigt de gender en diversiteit commissie jouw uit om deze enquête in te vullen.

Het duurt maximaal 5 minuten. Je mag je antwoorden ook in het Nederlands opschrijven.

De vragenlijst is anoniem!

Mocht je met iemand willen spreken over je ervaringen met intimidatie, dan kun je contact opnemen met een van de vertrouwenspersonen van de faculteit (Naam 1, telefoonnummer, email@adres; Naam 2, telefoonnummer, email@adres).

Bedankt voor je medewerking!

Namens de werkgroep harassment van de gender en diversiteit commissie
