# Supplementary material for: Underreported and unknown student harassment at the Faculty of Science
Source: PLoS One. 2019 Apr 25;14(4):e0215067. doi: 10.1371/journal.pone.0215067 (PMC6483172; doi:10.1371/journal.pone.0215067)
Supplement: S1 Table — (DOCX) [file pone.0215067.s004.docx]

**S1 Table** Number of years respondents have been studying

| Years | Count | Percentage |
| --- | --- | --- |
| 1 | 133 | 22% |
| 2 | 119 | 20% |
| 3 | 100 | 16% |
| 4 | 95 | 16% |
| 5 | 74 | 12% |
| 6 or more | 70 | 11% |
| Not completed | 19 | 3% |
