# Supplementary material for: Underreported and unknown student harassment at the Faculty of Science
Source: PLoS One. 2019 Apr 25;14(4):e0215067. doi: 10.1371/journal.pone.0215067 (PMC6483172; doi:10.1371/journal.pone.0215067)
Supplement: S2 Table — (DOCX) [file pone.0215067.s005.docx]

**S2 Table** Programs followed by respondents

| Program | Count | Percentage |
| --- | --- | --- |
| Bachelor Biologie | 110 | 18% |
| Bachelor Informatica | 66 | 11% |
| Bachelor Molecular Life Sciences | 47 | 8% |
| Bachelor Natuur- en Sterrenkunde | 62 | 10% |
| Bachelor Science | 22 | 4% |
| Bachelor Chemistry | 48 | 8% |
| Bachelor Wiskunde | 42 | 7% |
| Master Biology | 47 | 8% |
| Master Medical Biology | 45 | 7% |
| Master Molecular Life Sciences | 22 | 4% |
| Master Chemistry | 24 | 4% |
| Master Science | 13 | 2% |
| Master Physics and Astronomy | 25 | 4% |
| Master Mathematics | 20 | 3% |
| Master Computing Science | 32 | 5% |
| Master Information Sciences | 4 | 1% |
| Not completed | 33 | 5% |
