# Supplementary material for: Underreported and unknown student harassment at the Faculty of Science
Source: PLoS One. 2019 Apr 25;14(4):e0215067. doi: 10.1371/journal.pone.0215067 (PMC6483172; doi:10.1371/journal.pone.0215067)
Supplement: S3 Table — (DOCX) [file pone.0215067.s006.docx]

**S3 Table** Nationality of respondents

| Nationality | Count | Percentage |
| --- | --- | --- |
| Dutch | 481 | 78.9% |
| Not completed | 70 | 11.5% |
| German | 23 | 3.8% |
| Spanish | 5 | 0.8% |
| Greek | 3 | 0.5% |
| Dutch-German | 3 | 0.5% |
| Brazilian | 2 | 0.3% |
| Romanian | 2 | 0.3% |
| Other EU | 12 | 2.0% |
| Other non-Eu | 9 | 1.5% |
