# Supplementary material for: Underreported and unknown student harassment at the Faculty of Science
Source: PLoS One. 2019 Apr 25;14(4):e0215067. doi: 10.1371/journal.pone.0215067 (PMC6483172; doi:10.1371/journal.pone.0215067)
Supplement: S4 Table — (DOCX) [file pone.0215067.s007.docx]

**S4 Table** Experienced harassment of students by supervisors, students and other employees of the faculty.

| Experienced harassment | Supervisor | Students | Any other employee of the faculty |
| --- | --- | --- | --- |
| Once | 8 | 4 | 1 |
| Seldom | 4 | 8 | 3 |
| Regularly | 4 | 5 | 1 |
| Often | 3 |  | 2 |
| Not applicable | 8 | 9 | 17 |
| Not answered | 583 | 586 | 586 |
