# Supplementary material for: Underreported and unknown student harassment at the Faculty of Science
Source: PLoS One. 2019 Apr 25;14(4):e0215067. doi: 10.1371/journal.pone.0215067 (PMC6483172; doi:10.1371/journal.pone.0215067)
Supplement: S5 Table — (DOCX) [file pone.0215067.s008.docx]

**S5 Table** Observed harassment of students by supervisors, students and other employees of the faculty.

| Observed harassment | Supervisors | Students | Any other employee of the faculty |
| --- | --- | --- | --- |
| Once | 5 | 2 |  |
| Seldom | 7 | 12 | 2 |
| Regularly | 1 | 6 | 2 |
| Often | 2 | 1 | 1 |
| Not applicable | 13 | 8 | 19 |
| Not answered | 582 | 582 | 586 |
