# Supplementary material for: Underreported and unknown student harassment at the Faculty of Science
Source: PLoS One. 2019 Apr 25;14(4):e0215067. doi: 10.1371/journal.pone.0215067 (PMC6483172; doi:10.1371/journal.pone.0215067)
Supplement: S6 Table — (DOCX) [file pone.0215067.s009.docx]

**S6 Table** Experienced physical, psychological, verbal and sexual harassment according to the characteristics.

| Experienced harassment | Physical | | | Psychological | | | Verbal | | | Sexual | | |
| --- | --- | --- | --- | --- | --- | --- | --- | --- | --- | --- | --- | --- |
|  | Male | Female | Neither | Male | Female | Neither | Male | Female | Neither | Male | Female | Neither |
| My sex |  | 7 | 1 | 1 | 11 | 1 | 1 | 11 | 1 |  | 8 | 1 |
| My age |  | 1 |  |  | 2 |  |  | 2 |  |  |  |  |
| My skin color |  |  | 1 |  |  | 1 |  |  | 1 |  |  | 1 |
| My ethnic cultural origin |  | 1 | 1 |  | 1 | 1 |  | 1 | 1 |  |  | 1 |
| My physical appearance |  | 5 | 1 | 2 | 7 | 1 | 2 | 8 | 1 |  | 7 |  |
| My sexual orientation |  | 1 | 1 |  | 1 | 1 |  | 1 | 1 |  |  | 1 |
| My disability |  |  |  | 1 | 2 |  | 1 | 2 |  |  |  |  |
| The way I speak |  |  | 1 | 1 | 1 | 2 |  | 2 | 1 |  |  |  |
| My political ideas |  |  | 1 | 2 | 1 |  | 2 |  |  |  |  |  |
| My religion or philosophy |  |  | 1 | 1 | 1 | 1 | 1 | 1 | 1 |  |  |  |
| My social class | 1 |  | 1 |  | 1 | 1 |  | 1 | 1 |  |  |  |
| My native language |  |  | 1 |  |  | 1 |  |  | 1 |  |  |  |
| Unknown reasons |  |  |  | 4 | 1 |  | 4 | 2 |  |  | 1 |  |
| Other | 1 |  |  | 1 | 4 |  |  | 4 |  |  |  |  |
