# Supplementary material for: Underreported and unknown student harassment at the Faculty of Science
Source: PLoS One. 2019 Apr 25;14(4):e0215067. doi: 10.1371/journal.pone.0215067 (PMC6483172; doi:10.1371/journal.pone.0215067)
Supplement: S7 Table — (DOCX) [file pone.0215067.s010.docx]

**S7 Table** Observed physical, psychological, verbal and sexual harassment according to the characteristics.

| Observed harassment | Physical | | | Psychological | | | Verbal | | | Sexual | | |
| --- | --- | --- | --- | --- | --- | --- | --- | --- | --- | --- | --- | --- |
|  | Male | Female | Neither | Male | Female | Neither | Male | Female | Neither | Male | Female | Neither |
| Their sex | 1 | 4 |  | 6 | 6 | 1 | 5 | 7 |  | 4 | 6 |  |
| Their age |  | 2 |  |  | 2 |  |  | 2 |  |  | 1 |  |
| Their skin color |  | 1 |  |  | 1 |  | 1 |  |  |  | 1 |  |
| Their ethnic cultural origin |  | 1 |  |  | 2 |  | 1 | 2 |  |  | 1 |  |
| Their physical appearance |  | 4 |  | 2 | 7 |  | 2 | 7 |  |  | 5 |  |
| Their sexual orientation |  | 1 |  | 1 | 2 | 1 |  | 4 | 1 |  | 1 |  |
| Their disability |  | 1 |  |  | 4 |  |  | 4 |  |  |  | 1 |
| The way they speak |  | 1 |  |  | 3 |  |  | 4 | 1 |  |  |  |
| Their political ideas | 1 | 1 |  | 3 | 1 | 2 | 3 | 2 |  |  |  |  |
| Their religion or |  | 1 |  |  | 4 |  |  | 4 |  |  | 1 |  |
| philosophy |  |  |  |  |  |  |  |  |  |  |  |  |
| Their social class | 1 | 2 |  | 2 | 4 |  | 1 | 3 |  |  | 1 |  |
| Their native language |  | 1 |  |  |  |  |  | 1 |  |  |  |  |
| Unknown reasons | 1 | 3 | 1 |  | 1 |  | 1 | 1 |  | 2 | 3 |  |
| Other | 1 |  |  | 3 | 3 | 1 | 2 | 1 |  | 1 |  |  |
