# Supplementary material for: Underreported and unknown student harassment at the Faculty of Science
Source: PLoS One. 2019 Apr 25;14(4):e0215067. doi: 10.1371/journal.pone.0215067 (PMC6483172; doi:10.1371/journal.pone.0215067)
Supplement: S8 Table — (DOCX) [file pone.0215067.s011.docx]

**S8 Table** Examples of comments

| **Subject** | **Comment** |
| --- | --- |
| Physical other: | Social abilities |
| Psychological other: | My intelligence. |
| Psychological other: | For not completing my bachelor in 3 years, but choosing self-development and a board year. |
| Verbal other: | Not knowing things in the lab while these weren’t expected to know. |
| Verbal other: | Being a student (lower educated than the TA). |
| Experienced not reported: | It probably won’t make a difference and it is not easy to report somebody that you are depending on for a grade. |
| Experienced not reported: | I decided to fight this one myself. It was a rude other student, so I made it a personal discussion instead of an institutional one. |
| Observation not reported: | I don’t know where to report it and I find it not my business to report something for them. |
| Observation not reported: | The person it happened to, didn’t want that, so I respected that. |
